# Persistent Miscalibration for Low and High Achievers despite Practice Test Feedback in an Introductory Biology Course

**DOI:** 10.1128/jmbe.00139-21

**Published:** 2021-07-30

**Authors:** Jennifer L. Osterhage

**Affiliations:** a Department of Biology, University of Kentucky, Lexington, Kentucky, USA

**Keywords:** calibration, metacognition, Dunning-Kruger effect, testing effect, undergraduate biology

## Abstract

Students’ ability to accurately judge their knowledge is crucial for effective learning. However, students’ perception of their current knowledge is often misaligned with their actual performance. The relationship between learners’ perception of their performance and their actual performance on a task is defined as calibration. Previous studies have shown significant student miscalibration in an introductory biology course: students’ predicted exam scores were, on average, significantly higher than their actual scores. The goal of this study was to determine whether completion of a practice test before exams would result in better performance and calibration. The hypothesis was that students who completed a practice test would perform better and be better predictors of their performance on exams than students who did not engage in practice testing. As predicted, students who voluntarily completed a practice test, on average, performed better and were more calibrated than students who did not. Importantly, however, many of the lowest-performing students continued to significantly overestimate their knowledge, predicting higher scores on the exam than they actually earned, despite feedback from practice tests. In contrast, practice testing was associated with underconfidence in high-performing students. These findings indicate that practice tests may enhance calibration for many students. However, additional interventions may be required for the lowest-performing students to become better predictors of their performance.

## INTRODUCTION

Almost all undergraduate students enter introductory courses expecting to earn an “A” or “B” grade (1). However, gateway undergraduate science courses tend to have high failure (D/F) and withdrawal (W) rates (2), indicating that many students earn substantially lower grades than they initially anticipated. This incongruence has important consequences: science students may change their majors to a nonscience field because their grades in science courses did not match their expectations (3).

Metacognition, simply defined as the ability to think about one’s own thinking (4), consists of two key elements: metacognitive knowledge and metacognitive regulation (5). Metacognitive knowledge includes learning processes, awareness of effective learning strategies, and the ability to distinguish between knowing and not knowing. Metacognitive regulation refers to learners’ ability to accurately evaluate strengths and weaknesses, reflect on the success of their strategies, and adjust accordingly. Metacognitively aware students accurately assess a task, make plans, and effectively self-monitor during learning (6). For metacognitively unaware students, the perception of their current knowledge is often misaligned with their performance. When students do not grasp the limits of their understanding, they are at risk of underperformance and academic failure (7). Instructor strategies to promote student metacognition have shown promise, but intervention studies have lagged behind foundational research in this area (8).

### Calibration and the Dunning-Kruger effect

Calibration occurs when learners’ judgments are closely related to their actual performance on a task (9). Calibration measures have been used as indicators of metacognitive monitoring ability. There are multiple methods used to assess calibration, including calculation of the difference between predictions of performance and actual performance (10). Both over- and underestimates lead to miscalibration, the inaccuracy in judgment between perception of performance and actual performance (11). Judgment errors may influence study efforts, resulting in lower academic success (12). For example, overconfident students may prematurely cease study efforts when they believe that they have mastered concepts (13). Importantly, students who are poorly calibrated are more likely to earn lower course grades than calibrated students (14).

The least competent individuals are the most likely to be overconfident in their performance judgments. This cognitive bias, in which unskilled individuals are the most likely to overestimate their ability, is named the Dunning-Kruger effect (15). The Dunning-Kruger effect has been observed in multiple studies across various contexts (16–20) and has been shown to persist even when learners are presented with accurate information about their skill level (21–23). The Dunning-Kruger effect has been attributed to metacognitive differences between groups of learners. While skilled individuals are metacognitively aware, unskilled individuals have gaps or distortions in their knowledge that do not allow them to realize how unskilled they are (15).

Inaccurate performance judgments may persist as a self-protective mechanism. A qualitative analysis (24) observed distinct patterns of thinking for both high- and low-achieving students. High-achieving students reported underestimating themselves as to not appear immodest and to avoid disappointment. These students also used underconfidence as a motivational strategy to stimulate study efforts. Overconfident students, in contrast, were motivated by optimistic predictions of their performance. In agreement with this analysis, Helzer and Dunning (25) found that overconfident individuals gave more weight to their aspirations than evidence of past achievement when making performance judgments. Taken together, these studies suggest that both high- and low-achievement individuals use subjective measures rather than objective information to inform their performance judgments.

Overconfidence leads to the premature termination of studying and lower levels of retention (13). Given the high-stakes nature of exams in many undergraduate science courses, accurate judgments of preparedness for summative assessments are particularly important in these contexts. Therefore, inaccurate self-evaluation poses a significant risk for low grades in college science courses. In support of this assertion, in a first-semester chemistry course, the extent of overconfidence on a pretest predicted the likelihood of a failing final grade (26). In an introductory biology course, lower-achieving students had the most inaccurate estimates of their performance (27). In other studies of introductory biology and chemistry students, the lowest-performing students overestimated their exam performance, overall grades, and class rank (19, 28–30). These studies demonstrate that overconfidence is associated with lower course grades in introductory science courses, underscoring the important role of metacognitive awareness in these contexts.

### The testing effect (retrieval practice)

Taking tests during the learning process has been shown to lead to better long-term retention than other study methods such as rereading. The finding that testing is superior to restudying, called the testing effect or retrieval practice effect, is supported by robust empirical evidence (31–35). The contribution to long-term memory is not the only benefit of testing during learning: tests can also serve as a monitoring tool by giving students feedback about their level of understanding (36). Based on these findings, the use of testing as a learning strategy has been encouraged (31–37).

The use of testing for learning by students in undergraduate science courses has been described. In an undergraduate chemistry course, retrieval practice strategies were not as widely used as review-type strategies (38). In contrast, answering questions from old exams was the most popular study strategy for students in an introductory biology course (39). Students earning a “D” or “F” on the first exam in an introductory biology course reported lower usage of self-testing than higher-achieving students (40). Another study showed that self-testing increased over time among students in an introductory chemistry course (41). These studies highlight the importance of retrieval practice in college science courses.

### Retrieval practice and calibration

Because more information is available to inform performance judgments, it has been asserted that retrieval practice activities, followed by feedback about one’s performance, should enhance calibration (42). However, previous studies investigating the relationship between retrieval practice activities and calibration have yielded mixed results. Some studies support the assertion that learners have more accurate judgments after retrieval practice (43–45). However, practice testing was associated with greater miscalibration in other studies. For example, graduate students in a research methods course who completed practice tests were worse predictors of exam performance than students who did not (46). In another study, learners became increasingly underconfident with more practice across a variety of contexts (47). This effect was termed the underconfidence-with-practice (UWP) effect and has since been observed in multiple contexts (48–50). The conflicting reports about the nature of the relationship between practice testing and calibration suggest that feedback from practice has inconsistent effects on learners with different characteristics. Relatively little is known about how feedback from testing may differentially impact calibration for students with different levels of achievement.

Given the importance of accurate judgments of learning for academic success and the known benefits of testing, feedback from practice tests could be a particularly powerful tool to enhance success in science courses. The purpose of this study was to compare the predicted and actual exam performances of students who received feedback from a practice test with those of students who did not. The specific research questions were as follows:

1. Do practice tests enhance performance and calibration in an introductory biology course?
2. Do practice tests differentially affect calibration based on achievement level?

The hypothesis was that, on average, students who complete a practice test would perform better and be better predictors of their performance on exams than students who did not complete a practice test. Given previous research on the Dunning-Kruger and UWP effects, it was hypothesized that practice tests may have distinct effects based on achievement level. Specifically, the prediction was that low-achieving students would continue to be overconfident and that high-achieving students would become more underconfident after feedback from practice testing.

## METHODS

### Participants

Study participants were enrolled in Introductory Biology I at a 4-year public institution in the southeastern United States. Consenting participants (*n *= 341) were enrolled in one of four course sections during the Fall 2019 semester. All participants completed each exam in the course.

### Course description and setting

Introductory Biology I is a required course for the undergraduate biology major and other science and prehealth majors across the university. Contact hours consisted of 150 minutes per week throughout a 16-week semester. Topics covered included the nature of science, evolution, gene expression, cell division, inheritance, ecology, and biodiversity.

In the study semester, each of the four sections was taught by a different instructor. J. L. Osterhage was the instructor for one section. Learning outcomes, grading schemes, homework questions, practice tests, and actual exams were uniform across sections. The course schedule was the same across sections except for adjustments for class meeting patterns. All students were provided with the same course packet, which included section summaries, learning outcomes, practice questions, and study checklists. Instructors used different notes and practice questions during class time. Activities to promote self-evaluation were embedded throughout all sections: (i) clicker questions, which allowed students to see the percentage of classmates who chose each answer; (ii) group quizzing, in which students were encouraged to consider how many of their answers they changed after group discussion; and (iii) the availability of additional practice questions in the learning management system (LMS) and the course packet. Effective study strategies were discussed in class and included in the course packet. In all sections, students were encouraged, but not required, to complete the online practice tests described below. All instructors discussed how feedback from the practice tests should be used to identify areas of strength and weakness before the exam.

### Design and procedures. (i) Online practice tests

All students were provided with the same online practice test for each exam in the course LMS. Practice tests consisted of 45 to 60 multiple-choice questions used on exams from previous semesters, which were chosen to accurately represent the content of exams in the study semester. Except for the cumulative practice test, which contained 60 questions (compared to 86 questions on the final exam), the number of practice test questions matched the number of questions on the exam. The time limit of practice tests 1 to 3 was 75 min. The time limit for the final practice test was 120 min. In each case, the practice test time limit matched the time frame given for the exam. Practice tests were not proctored. Completion of the practice test was voluntary and did not affect the course grade. Correct answers were not visible until students submitted their answers. After submitting the practice test, students immediately received their score and could view correct and incorrect answers. No other feedback (e.g., explanations of answer choices) was provided.

### (ii) Exams

Exams were the same across sections and were completed during a common hour exam period. Students received a paper copy of the exam and filled in their answers on a Scantron sheet. The first and third exams consisted of 50 multiple-choice questions. The second exam consisted of 45 multiple-choice questions. The final exam was partially cumulative and consisted of 86 multiple-choice questions. No questions were duplicated exactly between the practice test and the actual exam, but there was considerable overlap between question styles and concepts tested.

The cover sheet of the exam included the statement, “Enter the numerical percentage (0–100) that you expect to earn on this exam _________.” Directly before beginning the exam, students were prompted to fill in the blank. Predictions of exam scores were gathered to capture students’ perceived levels of preparedness for exams before they occurred. After each exam, exam questions and answer keys were posted on the LMS.

### (iii) Data sources and analysis

Data collection and analysis were approved by the university’s institutional review board (IRB) (approval number 53301). Data were analyzed after final course grades were submitted for all students. A multiple-linear-regression model that included course instructor as a random effect was not significant (*P* = 0.9465) (data not shown). Therefore, data from all sections were combined for analysis.

All consenting students were included in the calculation of the performance quartile for each exam. Students who did not enter a predicted exam score were excluded from the calculation of discrepancy scores.

Discrepancy scores were calculated as the difference between students’ predicted and actual percentage scores on each exam (predicted score minus actual score). Positive raw discrepancy scores indicated that students overestimated their performance. Negative raw scores indicated that students underestimated their performance.

Student scores on the practice test were obtained from the LMS. Students were grouped into two categories: those who did not complete the practice test or scored ≤20% (the score expected for random guessing) (group A) and those who scored >20% (group B) on the practice test. The 20% cutoff was chosen because random guessing was not expected to have the same benefits for learning as an earnest effort. This approach was supported by one-way analysis of variance (ANOVA), which indicated no significant difference in exam performance and calibration between students who did not complete the practice test and those who scored ≤20%.

Mean discrepancy scores and mean exam scores were calculated for group A and group B and analyzed for each exam using Student’s *t* test. For group B, practice test scores were plotted against actual and expected exam scores. *R*^2^ was calculated for each scatterplot, and the relative strengths of correlations were compared using Fisher *r*-to-*z* transformation. Expected and actual scores were plotted against the actual percentile rank for each group. The area between the best-fit curves was highlighted to identify the relative contributions of each quartile to the overall discrepancy scores. Mean discrepancy scores were calculated separately for the lowest quartile and the highest quartile. To investigate the differences among means, data were analyzed using two-way ANOVA with the independent variables exam number and group and by three-way ANOVA including quartile as an additional variable. Pairwise comparisons of the means were performed *post hoc* using Tukey’s multiple-comparison tests. For each exam, Cohen’s *d* was calculated to measure the effect size. Effect sizes were averaged across exams to determine the mean effect size.

## RESULTS

### Do practice tests enhance performance and calibration in an introductory biology course?

**FIG 1:**
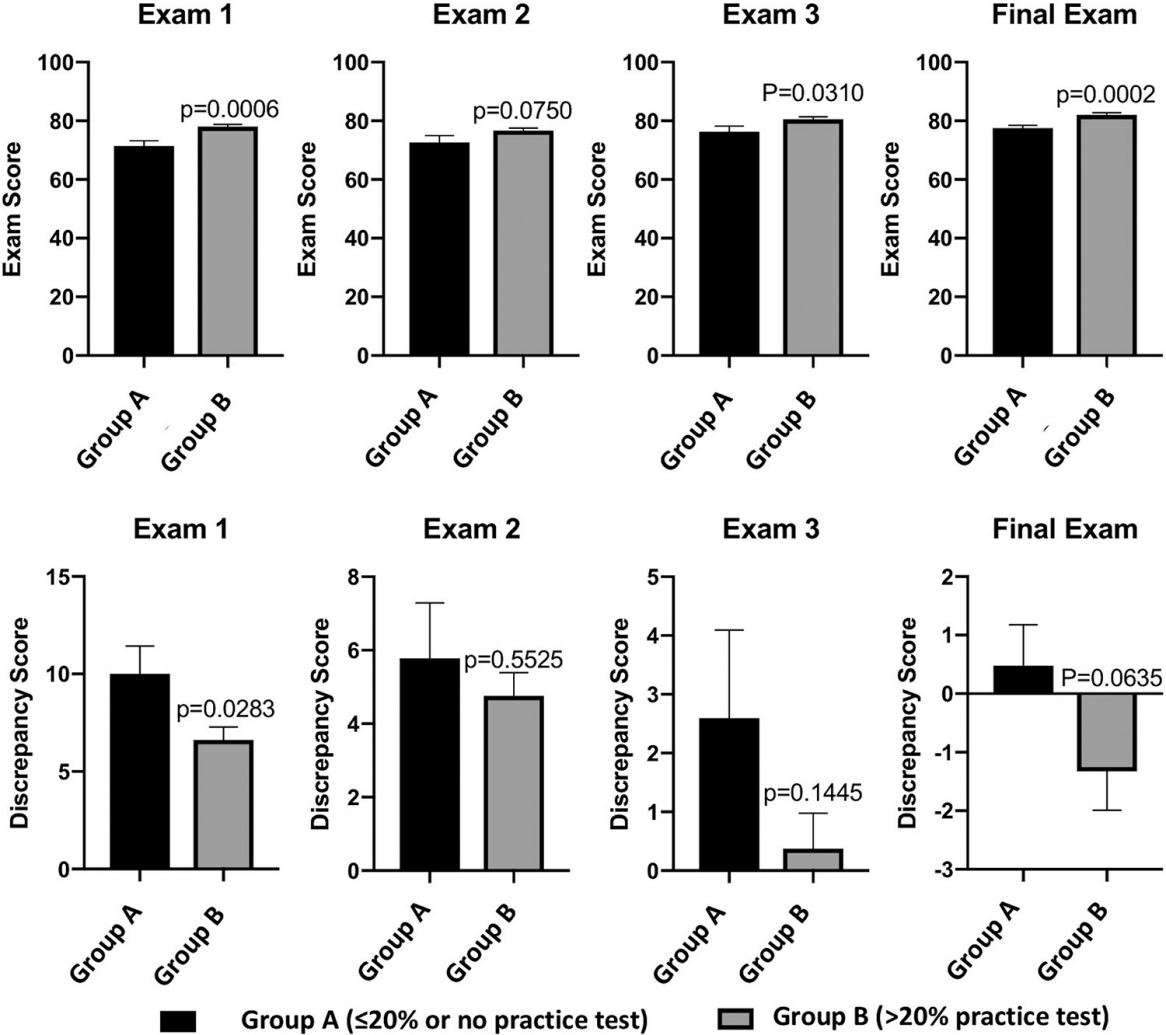
Completion of practice tests associated with improved exam performance and lower discrepancy scores. (Top) Students who completed the online practice test had significantly higher exam scores for exam 1, exam 3, and the final exam, with a trend toward improved performance on exam 2 (by a *t* test) (*n* = 341). (Bottom) Completion of the online practice exam with a score of over 20% (group B) was associated with significantly reduced discrepancies between the expected and actual earned scores of exam 1 compared with group A (by a *t* test) (*n* = 341). The trend was similar, although not statistically significant, for exam 2, exam 3, and the final exam.

The majority of students completed the practice test for exams 1 to 3, while fewer students completed the final practice test (Table 1). Consistent with the testing effect, students who completed the practice test (group B) earned significantly higher exam scores for exams 1, 3, and 4 than students who did not (group A) (Fig. 1, top, and Table 2). For exam 2, the difference in performances between groups A and B was not statistically significant. The effect size of practice testing on performance was greatest for the first exam (0.48), with a mean effect size across exams of 0.37.

**TABLE 1:** Percentages of students completing practice tests

Group (practice test score [%])	% of students who completed the practice test			
	Exam 1	Exam 2	Exam 3	Final exam
A (≤20 or no practice test)	18.5	12.8	16.8	45.4
B (>20)	81.5	87.2	83.2	54.6

**TABLE 2:** Mean exam percentages

Group (practice test score [%])	Mean exam score (%)			
	Exam 1	Exam 2	Exam 3	Final exam
A (≤20 or no practice test)	71.4	72.7	76.3	77.6
B (>20)	78.0	76.7	80.6	82.0

The degree to which students’ predicted scores were calibrated with their actual scores on exams was measured by calculating a discrepancy score, defined as the difference between students’ earned exam percentage and the percentage predicted before taking the exam. For exam 1, the mean discrepancy score for students who either did not complete the practice test or scored lower than 20% (group A) (mean = 10.0) was significantly higher than that for students who completed the practice test (group B) (mean = 6.6; effect size = 0.31) (Fig. 1, bottom left). The differences in discrepancy scores between student groups were not statistically significant for exams 2 to 4 (Fig. 1, bottom). Across exams, the mean effect size of practice tests on calibration was 0.21. For both groups A and B, discrepancy scores decreased as the semester progressed (compare the scales in Fig. 1, bottom).

**FIG 2:**
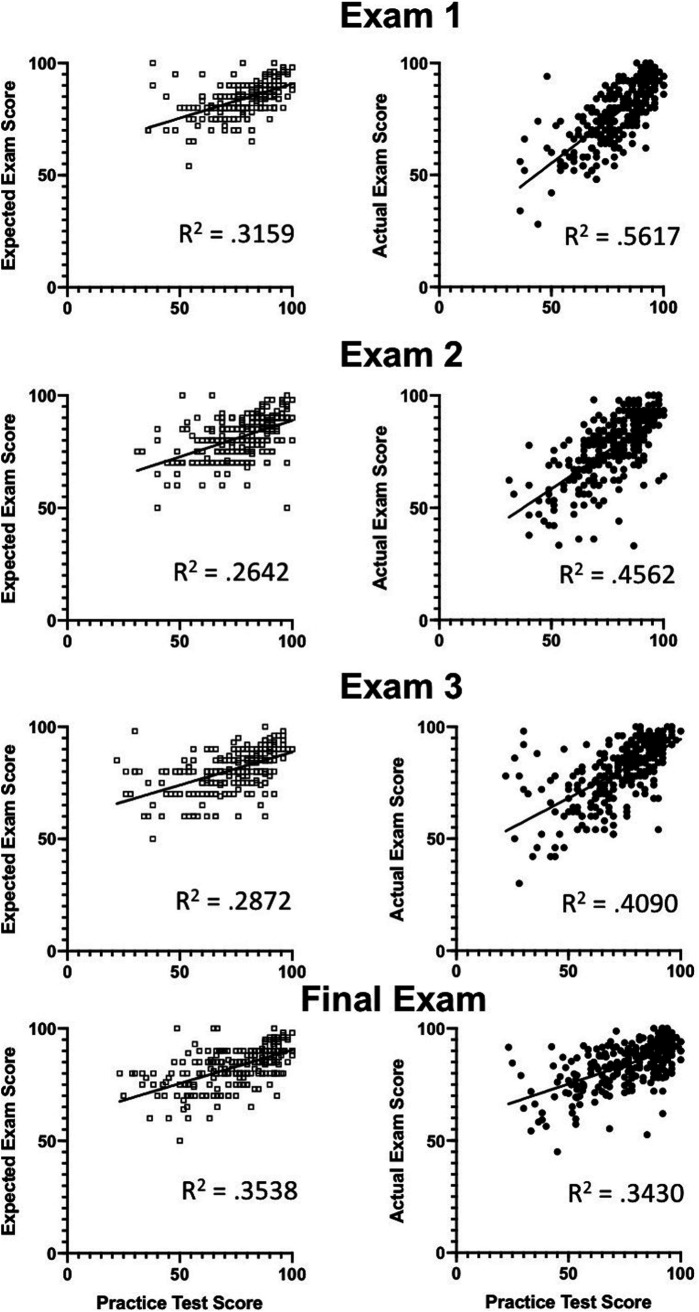
The practice test score is more highly correlated with the actual exam score than the expected score. For group B, practice test scores were plotted against expected exam scores (left) and actual exam scores (right). *R*^2^ was calculated for each scatterplot, and relative strengths of correlations were compared using Fisher *r*-to-*z* transformation.

Practice test scores were correlated with earned exam scores (Fig. 2, right), indicating that practice tests provided accurate feedback about the level of preparedness for exams. The correlation between student predictions of exam performance and actual performance, however, was less robust (Fig. 2, compare left and right panels). Even as average miscalibration decreased as the semester progressed, the correlation between practice test scores and expected scores did not change, indicating that feedback from the practice test was not the only factor that students used when making performance predictions.

### Do practice tests differentially affect calibration based on achievement level?

**FIG 3:**
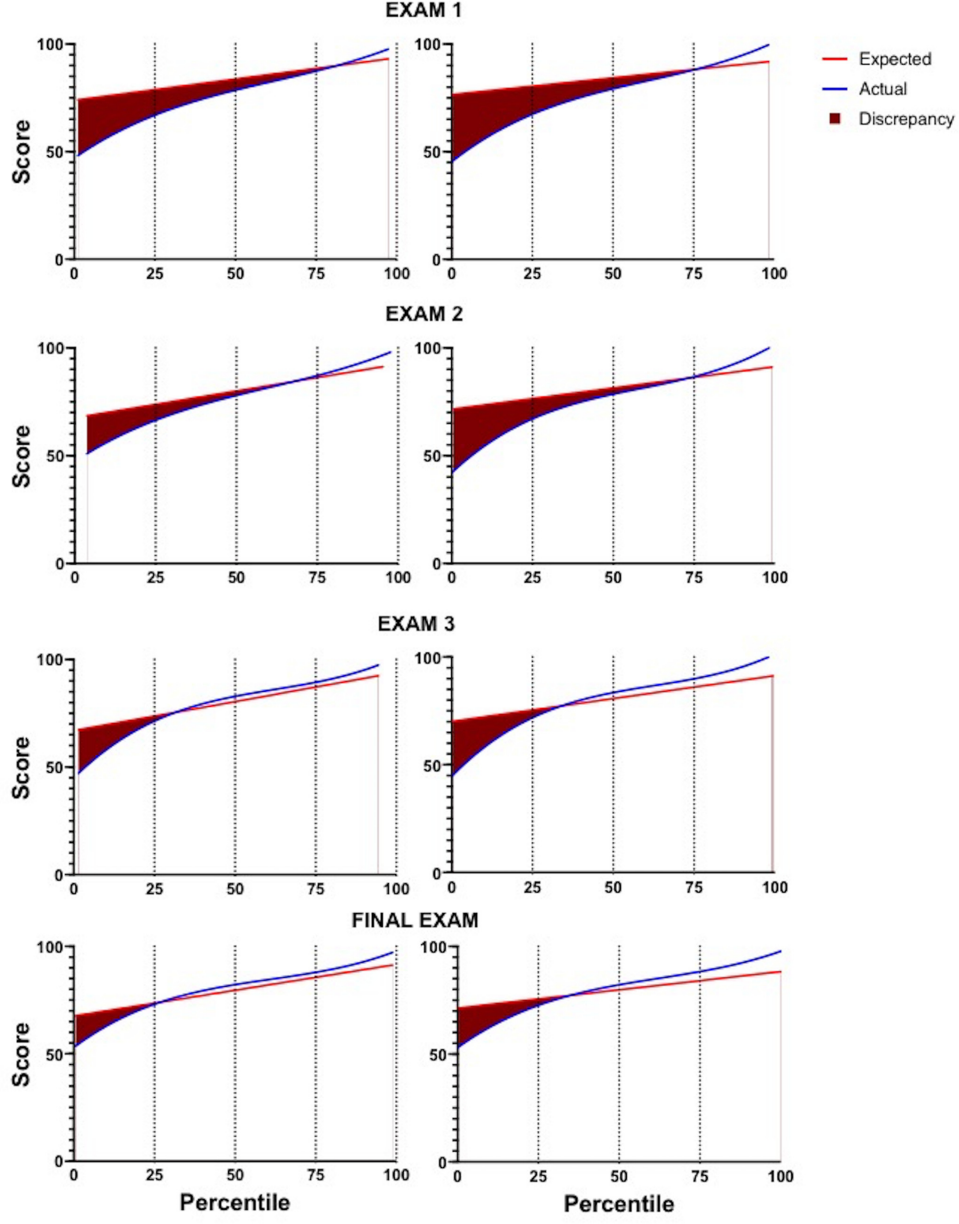
Distribution of expected scores and actual scores by percentile rank. Best-fit lines of predicted and actual scores graphed by percentile rank of actual scores were plotted for each exam. Expected scores and actual scores are depicted. The area between curves (dark red) represents overestimation. The majority of the discrepancy between actual and expected scores can be attributed to the lowest quartile.

Best-fit lines of actual and predicted exam scores were graphed against the percentile rank of performance. The Dunning-Kruger effect was observed whether or not students completed the practice test: the lowest-performing students were least calibrated when predicting their scores (Fig. 3, dark red). The correlation between actual and predicted exam scores increased as the semester progressed (Fig. 3, compare dark red areas among exams 1 to 4).

**FIG 4:**
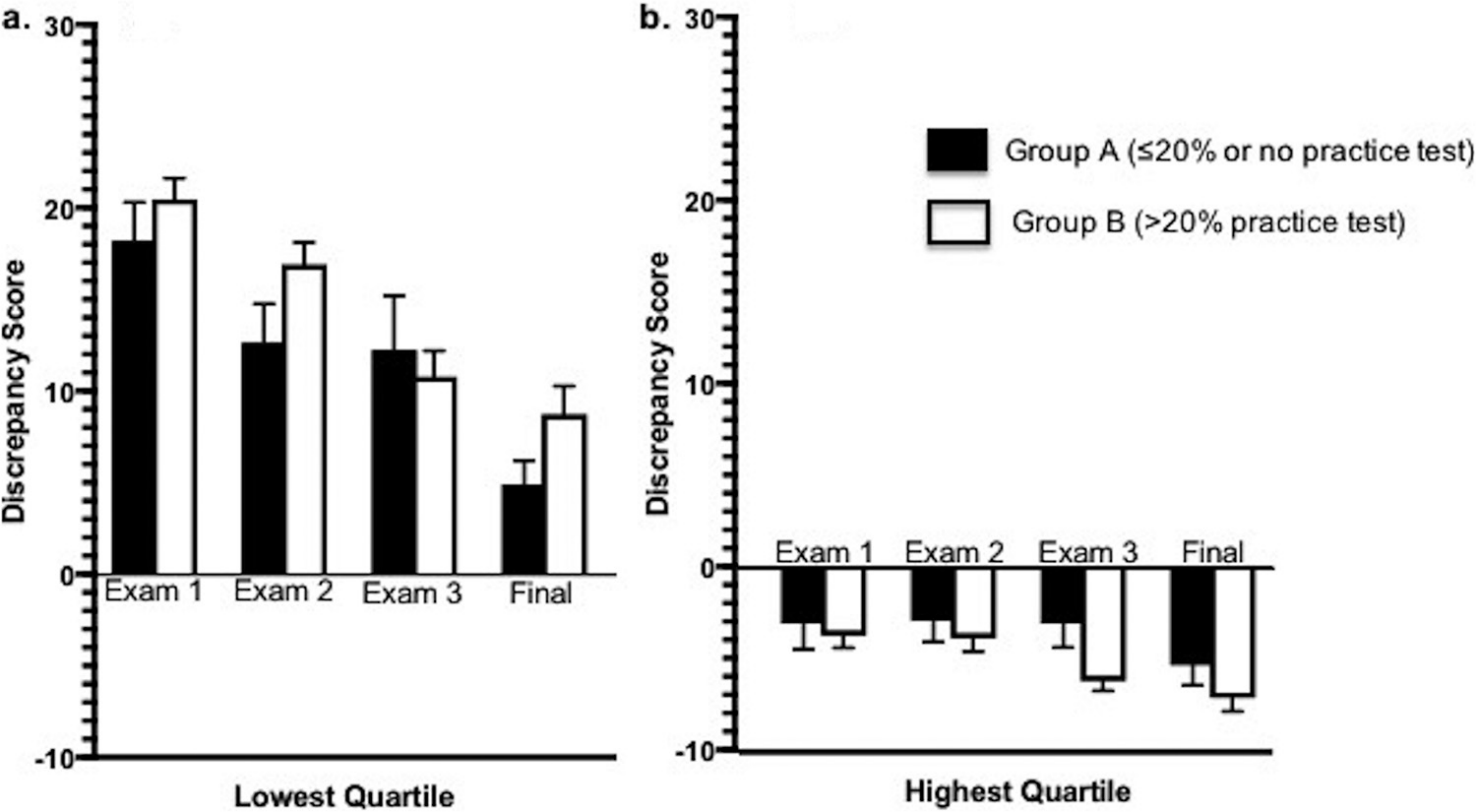
Practice exams do not lower miscalibration for the highest- and lowest-performing students. Discrepancy scores were plotted for the lowest quartile (a) and the highest quartile (b) based on exam score. (a) The lowest-performing students who completed the practice test trended toward more miscalibration than students who did not complete the practice test. (b) For the highest quartile, completion of the practice test was associated with underestimation of performance.

As a group, the lowest-performing students became less overconfident as the semester progressed (Fig. 4a, compare bar heights for exams 1 to 4). However, practice testing did not lower miscalibration for low-performing students (Fig. 4a, compare black and white bars). For the highest quartile of students, completion of the practice test was associated with greater underestimation of actual exam performance when all exams were analyzed together (*P* = 0.05; effect size = −0.34).

When grouping the lowest and highest quartiles of students across all exams, those who completed practice tests were significantly more miscalibrated than those who did not complete practice tests (*P* = 0.02). Taking the practice test exaggerated the tendency of both low- and high-achieving students to be miscalibrated albeit in different directions. Practice test completion did not lower overconfidence in low-achieving students and increased underconfidence in high-achieving students.

## DISCUSSION

Achievement in introductory science courses is negatively affected by miscalibration, a mismatch between performance judgment and actual performance. The central goal of this study was to determine whether feedback from practice testing improved performance and calibration of students in an introductory biology course. The data presented here are consistent with the testing effect, in which students who test themselves as a study strategy perform better on summative assessments. This finding was in agreement with a vast body of research on the benefits of testing for learning (for a review, see reference 37).

The relationship between practice testing and calibration was more complex. Early in the semester, students who completed the practice test were, on average, less miscalibrated than students who did not complete it. These results indicate that feedback, both from practice tests and from prior performance, contributed to enhanced calibration. While the overall impact of practice testing was better calibration coinciding with higher exam scores, a closer look at the distribution of expectations and performances revealed diverging effects on the highest and lowest quartiles. Practice testing did not mitigate the Dunning-Kruger effect, with many of the lowest-performing students continuing to predict much higher exam scores relative to their performance on the practice test (Fig. 2, left). The miscalibration of low-achieving students decreased throughout the semester, mainly due to better performance on exams, suggesting that practice testing did not significantly influence performance predictions. In contrast, high-achieving students underestimated their knowledge after practice testing and increasingly underpredicted their performance as the semester progressed. This may be the first study that has revealed a trend toward greater miscalibration for low- and high-performing students who complete practice tests than for those who do not.

Low-achieving students may use global self-concepts of academic ability rather than objective feedback to inform performance estimates (17). If that is the case, these students may require more feedback over a longer period to adjust their self-concepts. Reasoning ability is correlated with achievement in introductory biology (51). Low reasoning ability may affect both biology achievement and calibration because the skills and knowledge required to be successful in a discipline are the same ones required to assess one’s level of understanding (15).

Previous studies have demonstrated that the more practice an individual engages in, the more underconfident they become (the UWP effect). This study provides support for this effect, especially among high-performing students. The highest-performing students became increasingly underconfident relative to their abilities as the semester progressed, which was exacerbated by the completion of practice tests.

Many factors could contribute to the distinct effects of practice testing on calibration based on achievement level. The level of similarity between the practice test and the exam may differentially affect students based on their skill level. If the exam included many items that were not represented on the practice test, calibration could be negatively affected, especially for students with low understanding. In support of this hypothesis, students in an introductory biology course performed worse on new test items than on items that they were familiar with from old exams (39). In addition, a recent study demonstrated that performance on practice-tested items was significantly greater than that on nontested items (45). Even though students in this study were informed that the questions on practice tests would not be duplicated on exams, lower-performing students may have tried to memorize the answers to practice test questions and used their success at memorization as a basis for their predictions.

While not measured in this study, the amount and timing of retrieval practice activities may vary significantly between students. For example, a previous study indicated that the majority of students mass their studying the evening before an exam, which limits the use of effective study strategies (52). It is possible that students completed the practice test too close to the exam to affect their study strategies and calibration. Learners also tend to test themselves only under conditions that encourage retrieval success (36). It is possible that the lowest-performing students in the class engaged in fewer of the available alternative practice activities because they were not confident that they could be successful.

In agreement with a previous publication (27), in this study, average miscalibration decreased as the semester progressed. This finding suggests that feedback from prior exam performance informed predicted performance for later exams. The precise contributions of feedback from prior exam performance and that from practice testing were not measured. However, the significant difference in miscalibration between students who did and those who did not complete the practice test before exam 1 (Fig. 1) suggests that practice testing may be particularly important to mitigate miscalibration early in the semester, before other forms of feedback are available.

### Limitations and future directions

This study was limited in a few ways. First, it is possible that the learner characteristics of those who completed practice tests differed from those of students who did not. For example, students who completed the practice tests may have been more likely to believe that they could be successful (36). Students who completed practice tests could have had higher levels of metacognitive knowledge and regulation than others. Previous studies have shown that prior knowledge affects calibration when exam items were not previously tested by retrieval practice (45). In this study, prior knowledge was not measured, so it is not clear whether this may have affected calibration and exam performance. In addition, study strategy usage other than the practice test was not monitored in this study. It is possible that study strategies varied across the semester. For example, all previous exams were available for students to prepare for the final exam. The availability of these exams may explain why fewer students completed the practice test to prepare for the final exam. While no questions were exactly duplicated between the practice test and the actual exam, there were many questions on the practice test that required types of application and analysis similar to those required for the exam. This study did not track how many of the items on the practice test were directly comparable to exam questions. In addition, the timing of practice test completion and the amount of time spent engaging with the practice test were not tracked in this study. It is possible that some students took the practice test too close to the exam or did not engage with the practice test fully enough to reap the metacognitive benefits. In this study, group A was a heterogeneous population, consisting of students who did not open the practice test and those who earned a score of <20%. While it is not expected that random guessing or leaving most questions unanswered would confer cognitive or metacognitive benefits, these students did have access to the correct answers on the practice test. Therefore, it is possible that group A consisted of two fundamentally different subgroups. However, the level of engagement with the answer key could not be measured to identify any potential subpopulations.

In the present study, students were asked to predict their scores on exams directly before taking them as an indicator of their perceived level of preparedness. However, we cannot rule out that some students changed their predictions after taking the exam (postdiction). Future studies could compare prediction and postdiction performance estimates to determine if exam completion affects performance judgments.

In this study, feedback from the practice test consisted of the score earned and the ability to view correct and incorrect answers. It would be interesting to determine whether additional feedback, such as narrative descriptions of answer choices, would enhance the benefits of testing as a learning strategy. It would also be interesting to determine if these findings would apply to assessments other than multiple-choice-based exams. Future studies could also explore whether training about how to use practice testing as a study tool would enhance calibration and exam performance.

### Implications for instructors

The findings presented here suggest several instructor practices. First, providing full-length practice tests as a form of formative assessment may enhance overall student performance and calibration. Utilization of practice testing is particularly important for calibration early in the semester. Students may also benefit from explicit descriptions of the purpose of formative assessments and how to utilize the feedback. Instructors should keep in mind, however, that the use and effectiveness of this strategy will vary among students. Low-performing students may be resistant to changing their self-views and may require additional interventions to become better calibrated. If summative assessments represent a large portion of the students’ final grades, it will be especially important to reach miscalibrated students early in the semester, ideally before the first summative assessment. These strategies add to the existing toolkit (8) that instructors can utilize to foster student metacognition and enhance learning in undergraduate science courses.
